# ‘This big shadow that we need to turn into light’ – How labour intermediaries moralise commodified domestic care work

**DOI:** 10.1177/0308518X251377078

**Published:** 2025-10-14

**Authors:** Christiane Meyer-Habighorst, Christina Mittmasser, Karin Schwiter

**Affiliations:** Department of Geography, University of Zurich, Zurich, Switzerland; School for Social Work, University of Applied Sciences (HES-SO), Geneva, Switzerland; Department of Geography, University of Zurich, Zurich, Switzerland

**Keywords:** Domestic care work, labour market intermediaries, moralised markets, digital platforms, social entrepreneurship, visibility

## Abstract

Domestic care workers have long been largely invisible in labour statistics and in public and policy debates. The emergence of digital labour intermediaries has exposed domestic care workers to a new, but problematic, individual visibility: to find jobs and customers, they must create digital public profiles with personal information. Accordingly, scholars emphasise that this individualised visibility poses risks to privacy and security of domestic care workers. We argue in this paper that labour market intermediaries create not only an individualised visibility of domestic care workers, but also a new collective visibility that leads to more public debate about their working conditions and societal recognition. Methodologically, our argument is based on qualitative interviews with founders and managing directors of companies who mediate domestic care work in Switzerland. Drawing on the concept and literature on market moralisation, we consider these companies to be moral entrepreneurs. Based on our interest in how they frame the social value of commodified care work, we applied a discourse-analytic perspective. We found that the moral entrepreneurs use four central narratives to highlight their societal contribution. They claim to formalise care work, increase the public recognition of care work, emancipate women and integrate migrants. Based on these findings, we discuss these moralising narratives as ambivalent: although they reify a problematic feminisation, migrantisation and neoliberalisation of domestic care work, they enhance the collective visibility of care workers. Subsequently, the increased visibility might contribute to put working conditions in domestic care on the public agenda and lead to positive change.

## Introduction

In countries around the world, we can observe an accelerating care crisis (Fraser, 2017): On the one hand, ageing populations lead to an increase in the demand for care. On the other hand, austerity measures and retrenching welfare states, extended and unpredictable working hours as well as the need for two (or even more) incomes per household reduce its supply. These trends are amplified by a neoliberal notion of care as a private responsibility (Dowling, 2021). In combination, these developments lead to a two-tiered provision of care: working class households find themselves in an exacerbating domestic time squeeze. Well-off households turn to the market to fill their care gaps (Huws, 2019). The resulting commodification of care labour (Green and Lawson, 2011) turns households into workplaces for an increasing number of people – many of them women and many of them migrants (Cox, 2013; Griffiths and Santos Rodriguez, 2022; Kofman and Raghuram, 2015). This development is expedited by the rise of labour market intermediaries which facilitate the recruiting and placement of domestic workers for child and senior care, as well as for cleaning services. In recent years, they have become key players in shaping labour conditions as well as policy debates (Aulenbacher et al., 2024; Ledoux et al., 2021; Mercille and Lolich, 2024). Increasingly, these intermediaries rely on digital platforms to connect care workers and customers (Schwiter and Steiner, 2020).

The aim of this paper is to show how intermediaries for commodified domestic care work underline the positive impact of their companies on domestic care workers and society at large. Building on literature of ‘market moralisation’ (Balsiger, 2021; Shamir, 2008) and ‘social entrepreneurship’ (Bandinelli, 2017; Dey, 2010), we conceptualise them as *moral entrepreneurs*. This allows us to analyse how such actors moralise the domestic care market and to scrutinise the discursive effects of their moralising narratives. Thereby, we particularly focus on the issue of (in)visibility. Domestic care workers have long been largely invisible in public and policy debates (Hatton, 2017). The emergence of digital labour intermediaries has, however, exposed domestic care workers to a new individual visibility as they must create digital public profiles with personal information to find jobs and customers. Ticona and Mateescu (2018: 4384) put forward that this does not necessarily serve the interests of domestic care workers but rather poses risks to their privacy and security and thus exacerbates existing inequalities. In this paper, we argue that labour market intermediaries not only create a problematic ‘individualised visibility’ of domestic care workers, but also a novel *collective visibility* that leads to more public debate on their working conditions and recognition in society. Yet, this novel visibility also bares ambivalences, as it is embedded in an ever-evolving neoliberal agenda.

Empirically, our contribution is based on an analysis of the Swiss domestic care market. As the country with the highest wealth per adult globally (Credit Suisse, 2022), Switzerland is a particularly apt site to study the commodification of domestic care services. Not only does a considerable share of households have the means to buy care services, the country also ranks far below average among OECD countries with regard to its share of public spending for senior and child care services (Organisation for Economic Co-operation and Development [OECD], 2023; United Nations International Children’s Emergency Fund [UNICEF], 2021). Due to high purchasing power of customers and a generally business-friendly policy context (Schwiter et al., 2018), intermediaries such as digital labour platforms like to use the Swiss market as a testing ground for their services. Our analysis builds on 14 interviews and one public podcast with entrepreneurs who founded or manage a company that mediates domestic care work, many of them as digital platforms. Some of them offer domestic cleaning services, others focus on care and assistance for seniors, children or people with disabilities, others again are active in both fields.

## Commodified domestic care work, labour market intermediaries and (in)visibility

The rise of digital labour market intermediaries has heralded a further precarisation of labour in many fields of employment (Berg et al., 2018; Ticona, 2022). The effects on domestic care work, however, remain ambivalent. Already before the advent of digital platforms, it was a highly precarious and informal field of employment (Parreñas, 2001). As feminised work conducted in the space of the private household, it has received little attention by the public and policy makers alike (Strauss and Xu, 2018). Even though domestic care workers have a long tradition of organising and fighting for better working conditions (Ally, 2005) and feminist scholars and activists have strived for decades to put domestic care work on the political agenda (Hochschild, 1997), paid domestic care work is still characterised by insecurity and exploitation (Hunt and Machingura, 2016).

In paid domestic care work, where informality is the norm rather than the exception and labour policies do often not apply or are not enforced (International Labour Organization [ILO], 2013), intermediaries in many cases bring about a formalisation of labour (Huws, 2019). For example, digital platform interfaces can support workers in tracking hours worked and securing their earnings (Orth, 2024). Some of these intermediaries provide insurance for injuries sustained at work or damages to customer property (Hunt and Machingura, 2016). However, scholars emphasise that this formalisation typically remains selective. Intermediaries may formalise hiring and payment procedures and provide some social security benefits, but still eschew granting occupational protection and providing income security (Van Doorn, 2022).

Furthermore, up until today, domestic care work has been a particularly invisible sector of the labour market (Daniels, 1987; Hatton, 2017). Widespread informality means that a large proportion of domestic care workers is not represented in labour statistics. Workplaces in private homes further shield the workers from public view. Because they do not usually wear work clothes or other accessories that would identify them as domestic care workers, many domestic care workers tend to remain unnoticed when commuting from one household to another. This invisibility is also reflected in the literature on intermediaries like digital labour platforms that long focused nearly exclusively on the male-dominated field of delivery riders, whose fluorescent backpacks provided by the platforms make them much more visible in public space (Ecker et al., 2021).

The invisibility of domestic care workers does not only stem from informal work arrangements and the privacy of the workplace, but also from the lack of social recognition for the work itself and for those who do it (Hatton, 2017; McDonald et al., 2024). This devaluation is rooted in the intersection of capitalist and patriarchal structures, which lead to domestic care work being carried out predominantly by women for little or no pay (Fraser, 2017). Paid domestic work is thus mostly performed by migranticised and/or racialised women, resulting in a gendered and racialised division of work (Gutiérrez Rodriguez, 2011).

The COVID-19 pandemic temporarily made some domestic care workers (more child and senior care workers than cleaners) more visible as part of the essential workforce that had to continue their work when most other people had to (or had the privilege to) shelter in their own homes (Laugier, 2021; Pandey et al., 2021). While this moment fuelled a public debate on the value of care work, it did not (yet) induce a substantial revaluation of care (Schilliger et al., 2023). The devaluing of care work is thus not only present in the domestic sector but also present with regard to care homes for older people and children, a sector equally marked by processes of marketisation and corporatisation (Farris et al., 2024; Mercille, 2024).

Existing literature on digital care work platforms emphasises that labour market intermediaries impact the (in)visibility of domestic care workers (cf. Fetterolf, 2022; Gruszka and Böhm, 2022; Rodríguez-Modroño et al., 2022; Ticona and Mateescu, 2018). Especially in child and senior care work, algorithms show profiles to customers. This incentivises workers to share pictures and data about their private lives to find jobs. While this may provide the required visibility to access jobs, Ticona and Mateescu (2018) argue that it leads to a problematic ‘individualised visibility’ that exposes workers to the public eye and raises privacy and security issues. They conclude: ‘Ultimately, the visibility created by platforms does not realize the formalization of employment relationships, but does serve the interests of platform companies and clients and exacerbate existing inequalities for workers’ (Ticona and Mateescu, 2018: 4384). In this paper, we aim to contribute to this debate by arguing that labour market intermediaries not only create a problematic ‘individualised visibility’ but also a *collective visibility of* domestic care workers, their working conditions and value in society. To analyse the discourses of these actors, we draw from the literature on market moralisation presented in the following section.

## Conceptual approach: Market moralisation

While markets are always related to moral claims and orders, the process of market moralisation usually describes the emergence of ‘particular kinds of markets where explicit morality is the key principle of market interaction’ (Balsiger, 2021: 59). After decades of theorising and studying this process, many concepts have been applied to grasp this issue: ‘moral economy’ (Buttle, 2008; Wolford, 2005), ‘ethical consumerism’ (Ariztia et al., 2016; Barnett et al., 2011), ‘concerned markets’ (Geiger et al., 2014), ‘responsible capitalism’ (Bereni, 2023), ‘moralised markets’ (Balsiger, 2021; Suckert, 2018) and ‘social entrepreneurship’ (Bandinelli, 2017; Dey, 2010). Overall, they try to capture processes in which producer–consumer relationships are cast in ‘moral terms through a vocabulary of justice, partnership and solidarity’ (McEwan et al., 2017: 573).

The moralisation of markets usually concerns specific goods and services. Questions are raised, such as whether and under what conditions they should be exchanged within a market (and thus commodified). Often, this moralisation follows social movements in which consumers or producers criticise conventional market practices, for instance working conditions, and claim higher moral standards. Prominent examples include markets that deal with organic produce, fair trade products, animal rights and environmental or social justice issues (Balsiger and Schiller-Merkens, 2019).

In this paper, we analyse the moralisation of the domestic care market. In research on this particular market, morality is already a key concern, mostly discussed with regard to the intimate relationships between households as employers and domestic care workers as employees. Schilliger (2024), for example, draws on Folbre’s (2001) notion of the ‘prisoner of love dilemma’ to describe the strong sense of moral obligation of domestic care workers to their clients, which makes it difficult to resist precarious working conditions. Similarly, Näre (2011) argues that domestic work is based on a ‘moral economy’, on notions of good and bad beyond simple economic profit maximisation:

There is a general trend – on the part of the employers – to transform the contractual employment relationship into a moral contract, where it is out of gratitude, familial duty and affection that workers should perform their jobs and not for economic benefit. (Näre, 2011: 408)

In our analysis of morality within commodified domestic care work, however, we do not focus on the narratives of clients in private households, but on the labour market intermediaries that mediate domestic care services. We observe that those actors who found and manage these companies claim to improve the living and working conditions of domestic care workers. Therefore, we approach these intermediaries as *moral entrepreneurs*.

For a critical analysis of moralising narratives, we identify two aspects as crucial. The first is the performative nature of these narratives (Kish and Fairbairn, 2018; Kortelainen, 2008). As the moralisation of markets seems to be intensifying, it is increasingly criticised that social causes are only instrumentalised for marketing purposes (Millet, 2023). Debates around ‘green washing’, ‘pink washing’ and ‘diversity washing’ illustrate the growing concern about whether moralised markets are actually moralising capitalism or rather capitalising on morality (Bosvieux-Onyekwelu and Boussard, 2022). The moralisation of markets may, in other words, result in a sort of feel-good entrepreneurialism that in fact does nothing to address underlying societal and environmental issues (Kelpin, 2020). Simultaneously, Zelizer (1979) compellingly shows in her study of life insurance in the United States that moral discourse can influence and shape market development over time. She illustrates how public opinion about markets is shaped by narratives promoted by actors like companies and the media, revealing the strong ties between markets and moral values. This debate, whether morality is primarily a promotional and self-branding measure by businesses, or whether it indeed illustrates a growing public demand for ‘social consciousness’ (Arvidsson, 2014; Bandinelli, 2017) is still ongoing. While it is rarely either/or, the existing literature emphasises the limits of moralised markets, as they might ultimately ‘not withstand the forces of the market’, for example in terms of price competition (Balsiger, 2021: 60).

The second crucial aspect in critically analysing moralising narratives is understanding market moralisation as part of neoliberalisation. Neoliberalism is a political theory that has gained traction from the 1970s onwards with the promise of fixing the ails of the crisis-prone capitalist system (Harvey, 2005). The neoliberal rationale propagates freeing capitalism by rolling-back most state regulations and strengthening individual responsibility, competitive markets and private enterprise to foster economic growth (Brenner et al., 2010). Neoliberalisation then refers to historically and geographically variegated and politically guided processes of implementing this free-market rationale (Peck and Tickell, 2002). The moralisation of markets aligns with this rationale by using market-based approaches to tackle social issues. It offers alternative solutions to welfare state programmes, focusing on innovation and efficiency of markets rather than traditional government-led approaches (Nadai and Canonica, 2019, see also: Rosenman et al., 2024). In this regard, Shamir (2008), argues that the moralisation of markets within neoliberalism is not an external correction to economisation but rather a product of it. Facilitated through business education, academic discourse and organisational incentives, governments delegate socio-moral tasks to market entities. Businesses then handle tasks once considered as the responsibility of the welfare state, blending social goods with profit-seeking. Many scholars draw on Michel Foucault’s work on the ‘entrepreneur of the self’ and ‘neoliberal governmentality’ (Foucault, 1988, 1991) to critically discuss social entrepreneurship as a ‘government technique’ for individualising responsibility (Dey, 2014).

We follow this literature by tackling the ambivalent nature of the moralisation of markets: On the one hand, it makes certain societal issues and market practices visible and signals the desire for change. On the other hand, it ‘hijacks ethical feelings towards economic actions’ (Bandinelli, 2017: 23) and follows a neoliberal logic which does no longer hold the state responsible for the welfare of its people (Dey, 2010: 2). With regard to care work, this translates into governments replacing public child and senior care facilities by cash for care systems that incentivise families to buy care from market actors (Schwiter et al., 2018). In respect of domestic cleaning, this process of responsibilisation is less straightforward as cleaning has always been regarded as a private task rather than the responsibility of the state. Here, it is the domestic time-squeeze resulting from the need for more paid hours to make ends meet that pushes households to hire domestic workers (Huws, 2019).

## Methodology: Analysing the narratives of moral entrepreneurs

Our data consists of 14 interviews with entrepreneurs who mediate domestic care work in Switzerland. Within this small sample, we aimed at capturing the diversity of labour market intermediaries in the domestic care market according to the theoretical sampling approach suggested by Glaser and Strauss (1967: 45). The sample includes for-profit companies, non-profit associations and cooperatives. It contains small-scale local initiatives, larger national companies and international companies, all operating in German-speaking Switzerland. While a couple use conventional websites and web forms to interact with customers, the majority operates as digital labour platforms that include on-demand, marketplace and digital placement formats (see Rathi and Tandon, 2021: 14ff for a detailed explanation of the different digital platform types). Some entrepreneurs employ domestic care workers directly via their company; others only mediate between workers and households. With one exception, the entrepreneurs we interviewed are all (co-)founders or managing directors.

The interviews took place between spring and fall 2023 as part of two different research projects. The first is a trinational project on urban platform economies in care services.^1^ The second is a national research project examining the effects of the Covid-19 pandemic on domestic workers in Switzerland.^2^ Although the aims of these two projects vary, they share a common interest in intermediaries within the domestic work sector in Switzerland. Both used a semi-structured, problem-centred expert interview approach (Döringer, 2020). The interviews were conducted in Swiss German, German and English and lasted between 45 minutes and 2.5 hours. We complemented the 14 interviews with a public podcast interview with the founder of one of the most prominent digital platforms in the domestic care sector in Switzerland, Batmaid^3^ (Swisspreneur, 2023). Finally, we supplemented the interviewed data with material obtained from the websites of the 15 intermediaries.

For the analysis, we employed a discourse-analytical approach based on Waitt (2016). It entails ‘turning the gaze’ towards narratives, framings, legitimation strategies and their underlying power relations. This type of discourse analysis helped us explore how discourses shape our perception of specific issues (Hall, 2001). For this, we transcribed the conducted interviews verbatim with the support of transcription software and a research assistant.^4^ Subsequently, we used the software MAXQDA for descriptive and analytical coding. Our coding process was both inductive and deductive, meaning that certain codes emerged directly from the data while others were inspired by other studies on domestic care work, labour market intermediaries and the moralisation of markets. Following Waitt (2016), we paid particular attention to discursive patterns that appeared repeatedly and arguments that were presented as self-evident and unproblematic.

In line with our discourse analysis, our data does not indicate how the companies examined operate in practice (for an overview on this see e.g. Rodríguez-Modroño et al., 2022). Accordingly, we do not draw conclusions about their actual working practices. Instead, our analysis focuses on the verbal and written strategies employed by moral entrepreneurs to present their companies. Consistent with this discourse-analytical approach, we present and interpret these narratives and critically reflect on their implications.

## Findings: The moralising narratives of commodified care entrepreneurs

To show how entrepreneurs moralise the domestic care market in Switzerland, we analysed the narratives of our interviewees and identified four key ways in which the moral entrepreneurs talked about their work and companies. We argue that they claim to (1) formalise domestic care work, (2) increase the recognition of domestic care work, (3) promote the emancipation of female workers, and (4) support the integration of migrant workers. Thereby, they try to underline the moral value of their companies, but also engage in broader debates on commodified care work.

### Formalising domestic care work

First, the entrepreneurs interviewed address the fact that domestic care work has historically been, and often still is, informal, by claiming that their companies formalise employment relationships between workers and households. This narrative is particularly striking in the example of Batmaid. Founded in 2015, the Swiss digital platform for domestic cleaning services now operates in 6 Central European countries and employs more than 4000 cleaners across Switzerland (Batmaid, 2024). On their website, the digital platform summarises their aim as follows: ‘Providing declared and insured employment for people in the cleaning industry has always been essential to us, and we are actively engaged in combating undeclared work’ (Batmaid, 2024). Even the name of the platform was chosen accordingly, as the founder elaborated in the podcast interview:

I mean Batmaid, heroes from the shadow [. . .]. It’s not only the shadow that our clients [are] almost never at home when the cleaning lady or the cleaning man pass to do the cleaning but it’s more about the informal market. It’s the hero that works in this big shadow that we need to turn into light. And this was our goal to put this name on it. (intermediary 15)

**Figure 1.**
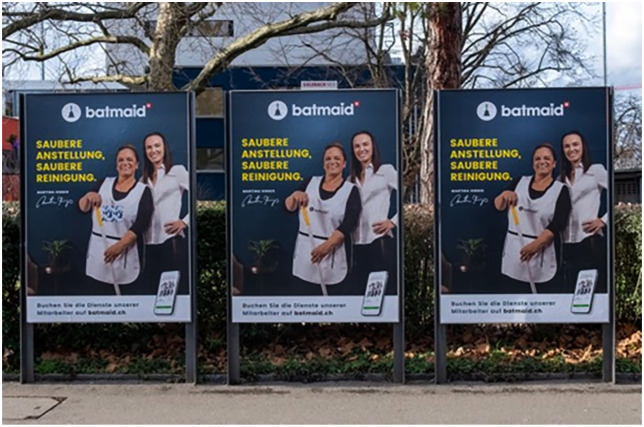
Marketing campaign of Batmaid under the slogan ‘Clean employment, clean cleaning’ (Beatrice Geistlich, Wochenzeitung [WOZ], 2024).

The founder further explained that he sees actively educating both workers and society about labour rights as the most efficient approach to formalisation. The narrative of formalisation is also very visible in Batmaid’s marketing campaign. Posters with their slogan ‘Clean employment, clean cleaning’ can be seen in many Swiss cities (see Figure 1).

In a similar way, many of our interviewees used the narrative of formalisation to highlight the added social value of their companies. They underlined that their aim is to ‘get away from informal labour’ (i. 7) and that they ‘do not tolerate informal work’ (i. 2).^5^ They thus emphasised their compliance with formal employment conditions, such as the existence of employment contracts, insurance or continued payment of wages in case of sickness and vacations and they argue that these directly benefit the workers. One interviewee, for example, pointed out that ‘everything is regulated. They [the workers] have insurance. It’s officially paid for’ (i. 12). Similarly, another interview partner underlined that workers are insured: ‘even if they [the childminders]^6^ fall ill or are absent due to illness [. . .] they still receive 80% of their salary for five days and are then actually covered as normal, just as any other employee’ (i. 1). Some interviewees even highlighted that they directly employ the workers and do not leave these responsibilities to the households. The narrative of formalisation is thereby at the core of the moralising narratives we find in our interviews with entrepreneurs in the domestic care market.

### Increasing recognition for domestic care work

Second, the entrepreneurs we interviewed address the devaluation of domestic care work and its general lack of social recognition. Accordingly, they present the fight for more societal recognition and appreciation of domestic care work as one of the core objectives of their companies. This narrative is particularly promoted by Good Work, a social business for cleaning services. Its website highlights that the business is committed to ‘the appreciation of domestic and cleaning work, [. . .] legal and fair employment [. . . and] a decent wage’ (i. 11). In the interview, the founder explained that the lack of appreciation is the most striking issue in the sector and thus directly linked to precarious working conditions and inadequate wages. The founder made this argument by comparing customers’ willingness to pay for cleaning work compared to traditionally male-dominated services:

The biggest shortcoming in the whole context is really this lack of appreciation for this work. Quite a lot of customers who want to purchase this service, who also want to externalize this work, are ultimately not prepared to pay a reasonable price for it. And because this willingness is lacking and we are also subject to strict limits. Our customers now pay between 45 and 50 [Swiss] francs per hour. And a lot of people consider that to be a lot. [. . .] I think appreciation is the first problem. Yes, everything derives a bit from that and that is both the way domestic workers are often treated and how they are invisibilised. In my view, it all stems a bit from this poor appreciation and, of course, the very poor working conditions in many places, because it’s really difficult to finance more here. [. . .] We pay an hourly wage of 120 [Swiss] francs for electricians. We probably pay 100 for plumbers and 80 for gardeners and the willingness to pay for these services is completely skewed when you compare that to domestic workers. (i. 11)

The narrative of increasing the recognition of domestic care work is echoed by numerous other interviewees. They thus often link the appreciation for domestic care work to adequate pay. One entrepreneur, for example, underlined that their company offers a ‘professional service’ and ‘nobody is employed as a hobby’, emphasising that ‘a person working in care work should have the opportunity to earn a good wage with good conditions’ (i. 7). Another interviewee put forward: ‘We actually hope to be able to establish the profession [of childminders] a little better and that it will also be financially worthwhile in the end’ (i. 1). In this sense, interviewees claimed that their companies will increase the societal recognition and appreciation for domestic care work.

### Emancipating female workers

The third narrative we identified in our interviews concerns the issue of gender equality. The entrepreneurs claim that their businesses foster the emancipation of women. The intermediary Mommys at Work is an illustrative example in this regard. It offers various services such as cleaning, catering and care for children, seniors and pets. It specifically targets mothers, not only as clients but also as workers. Thereby, the founder and managing director emphasised that they ‘always [. . .] focus on empowering women working in these areas’ (i. 7). The Mommys at Work website claims that the company

gives women the opportunity to pursue a flexible and freely adaptable employment opportunity with very good working conditions. In contrast to other providers on the market, we openly declare our prices and wages, and our mums can choose their workload and working hours themselves. (i. 7)

Many other entrepreneurs also use the narrative of emancipation to underline the added social value of their businesses. They see the flexible and part-time character of commodified domestic care work as a key aspect thereof, as it allows workers to combine private care responsibilities with gaining financial independence (cf. James, 2024). One interviewee underlined that flexible working hours ‘make it easier to reconcile family and career’ (i. 9). Similarly, another interviewee pointed out that domestic care work can be an opportunity for women who have stayed at home full-time with their children and want to ‘get involved again’ in paid work (i. 2).

Additionally, the interviewed entrepreneurs aim to support female workers by providing further training and education. This was particularly salient in our two interviews with cleaning cooperatives. The cooperatives were created by domestic care workers themselves with the aim to not only work in private households, but also to gain experience as entrepreneurs and managers. One representative directly linked this to the feminist cause: ‘The cooperative is very feminist in principle. It is primarily about the advancement of women, the emancipation of women, empowerment of women’ (i. 8).

### Integrating migrant workers

**Figure 2.**
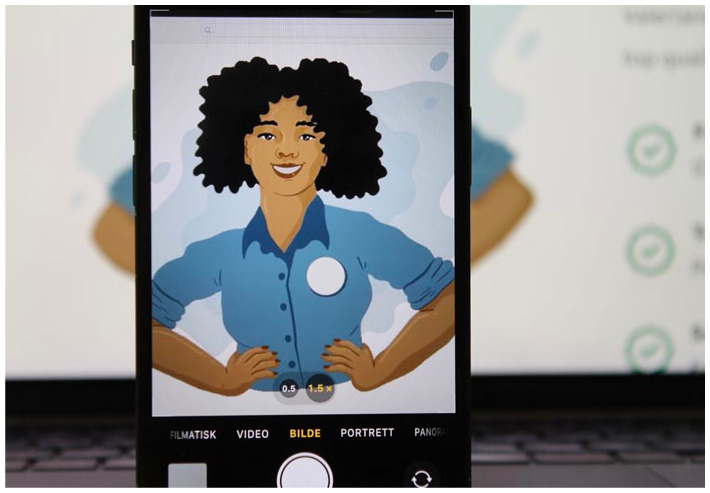
Presentation of a worker on the website of Amelia (photograph taken by authors 2025; badge whitened for the sake of anonymisation).

**Figure 3.**
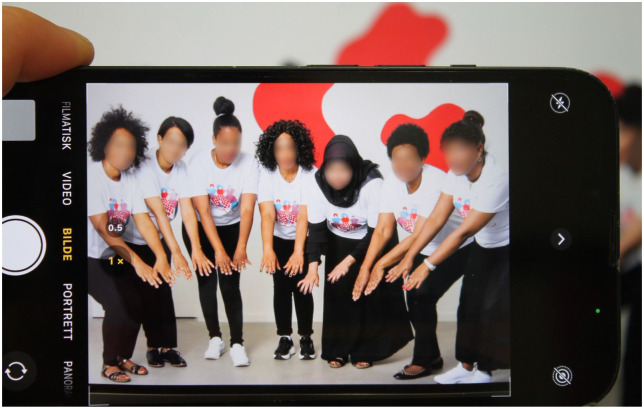
Presentation of workers on the website of Amelia (photograph taken by authors 2025; faces blurred for the sake of anonymisation).

Lastly, the interviewees often specifically underline the integration of migrants as an added social benefit of their companies. This narrative serves, for example, as the central motive of the non-profit business Amelia. In the visual representations of the company, women of colour are put centre stage (see Figures 2 and 3). On their website, Amelia advertises their cleaning services with the slogan:

Doing good – for yourself and others. Are you looking for someone to help you with your chores? Would you like to give back and support people with a migration background to integrate into Swiss society at the same time? Then you’ve come to the right place! (i. 9)

In the interview, the co-founder of Amelia argued that the three aspects ‘work, education, community’ (i. 9) are key to integration, and their company provides all three of them. ‘Getting ahead, learning, being reliable, doing a good job’ are thus ‘an integral part of the contract’ when working for Amelia (i. 9). The co-founder further explained:

There are certainly more women [. . .] who would be grateful to earn something on the side for a fair wage, to support the family and to be a role model for the children. It also makes it easier for the children if the mother is in touch with local people, if she has to speak German, improve her German. [. . .] After all, we are nudging people towards happiness. (i. 9)

Although not all our interviewees refer to integration as one of their main objectives, this narrative is also visible in other interviews. One interviewee pointed out: ‘it’s not just about domestic help, but about friendship, about relationships, about language’ (i. 12). In this spirit, several of the intermediaries presented in this paper, such as Mommys at Work, Amelia and the two cleaning cooperatives, also offer German classes to their workers. Some even hold workshops that aim to prepare them for the Swiss labour market.

## Discussion: Ambivalences of moralising the domestic care market

Conceptualising commodified domestic care work as a moralised market allows us to highlight the multiple ways in which entrepreneurs operating in the sector of domestic care work frame the added social value of their companies. In what follows, we reflect on the effects of these narratives and discuss the moralising narratives of entrepreneurs as an ambivalent undertaking.

Our key argument is that the presented moralising narratives lead to greater societal visibility of domestic care workers. The fact that labour market intermediaries position themselves as moral entrepreneurs, claiming to improve the living and working conditions in commodified care work, makes domestic care workers more visible in public space. They appear prominently in companies’ slogans and advertising campaigns, on their websites and in the narratives of their founders and managing directors. We therefore argue that these labour market intermediaries do not only create a problematic individualised visibility of workers on digital platforms, as put forward by Ticona and Mateescu (2018), but at the same time make them collectively visible as a social group with specific needs.

This collective visibility can lead to a growing sensitisation in society and foster public debate on decent working conditions and adequate pay for domestic care work. In Switzerland, this is reflected in recent heightened media interest across the major news portals (Basellandschaftliche Zeitung [BZ], 2021; Blick, 2020, 2021; Neue Zürcher Zeitung [NZZ], 2020, 2021; SRF, 2022; Tages-Anzeiger, 2022, 2024; WOZ, 2022, 2024). In addition, some of our interviewees argue that the public debate on domestic care services has led to an increasing demand for better working conditions on the side of clients. While our interview data provides no evidence as to whether the claims made by the moral entrepreneurs are actually realised in practice, we argue, in line with Bandinelli (2017: 23), that moralising narratives ‘still signal the attempt and desire to express and actualise ethical feelings and responsibility’ and thus impact public discourse. Following Zelizer (1979), this impact on public opinion through moral discourse can, in turn, contribute to changes in the domestic care work market in the long run. Furthermore, the narratives of labour market intermediaries may strengthen already existing political campaigns led by workers. The Swiss network *Care Work Unite*, for example, was founded in 2021 to improve working conditions and societal recognition in institutionalised as well as domestic care. They visibilise the demands of care workers in public rallies such as the Labour Day protests and the yearly Swiss feminist strike on June 14th.

While we argue that the narratives of moral entrepreneurs contribute to a collective visibility of domestic care workers, we are also aware of their ambivalent character. The moralisation of this market is certainly intensifying but so might be the extent to which social causes are instrumentalised for marketing purposes. Similar to what other authors have discussed for other sectors (e.g. Kelpin, 2020; Millet, 2023), we also need to question whether the moralisation of commodified domestic care work only results in a sort of feel-good entrepreneurialism that does not really address and tackle the underlying issues of decent work and might even exacerbate others. Our analysis shows that several aspects of commodified domestic care work are not problematised or even reified in the moralising narratives of entrepreneurs, namely the feminisation, migrantisation and neoliberalisation of commodified domestic care work.

First, we find that the moralising narrative of our interviewees on emancipation often builds on stereotypical representations of women. They are portrayed as ‘women in need of help’ – help with returning to the labour market, with balancing care and career and with improving their financial independence. This discourse is problematic in itself, as it implies that it is the women who need to be fixed rather than the (capitalist and patriarchal) system. Furthermore, it hides the fact that even when (partially) formalised, domestic care work rarely pays a living wage (Macdonald et al., 2018; McDonald et al., 2024), and it typically does not serve as a stepping stone to higher paid work. Rather, women often remain in the sector and rarely find options for further training and career development (Van Doorn, 2023). With their moralising narratives, our interviewees do not challenge the feminisation of domestic care work. On the contrary, they continue to channel women into this field of precarious work.

Secondly, we need to critically reflect the narrative of integration. The ways in which some of our interviewees use this concept reproduce social hierarchies between ‘locals’ and ‘migrants’ rather than breaking them up. Instead of questioning the racialised distribution of work in our society, migrants are presented as ideal for the job and portrayed as people who need further education and training. This narrative portrays migrants as people who need to prove their ability to contribute to the economy and achieve independence without state support (for Switzerland see e.g. Bachmann, 2016; Mexi et al., 2021; Mittmasser and Stingl, 2021). In this, it reproduces a neoliberal and neocolonial understanding of society that frames integration as the individual duty of ‘migrant others’ and reinforces the normalcy and superiority of those who were there before (Schinkel, 2018). These connotations are also visible in the narratives of certain moral entrepreneurs.

The moral narratives of integration and emancipation both resonate with Näre’s (2011) understanding of a ‘moral contract’ applying to domestic care workers. She shows that ‘the moral economy of domestic and care work is based on notions of duty, gratitude, altruism and familial responsibility’ (Näre, 2011: 407). While she refers to the relationship between care workers and their employing households, we find a comparable moral economy in the narratives of our labour market intermediaries. They suggest a ‘moral contract’ based on the promise of integration and emancipation for the workers. This focus on the potential integrative and emancipative benefits for care workers can be problematic if it is used to justify or draw attention away from the continuation of precarious working conditions and low pay in the sector.

Finally, our contribution serves as evidence of how morality is increasingly economised by businesses who claim to work for the societal good rather than just for their shareholders (Dey, 2014; Millet, 2023). This can be problematised as part of an ever-evolving neoliberal agenda, in which the market handles tasks once considered as the responsibility of the welfare state, blending social goods with profit-seeking. This is particularly evident in the case of childcare (and less so in the context of domestic cleaning work), where Switzerland remains one of the countries with the highest childcare costs relative to household income (OECD, 2025). Furthermore, an increasing share of subsidies are flowing towards private providers of childcare, such as family daycare and childminders (Bundesamt für Sozialversicherungen [BSV], 2025). This ‘responsibilization of market entities to assume the caring and welfare moral duties’ (Shamir, 2008: 10) is problematic in the context of domestic care work as care is reframed as a commodified good that needs to be purchased in the market rather than a public service provided by the state (Fraser, 2017). As Huws (2019) points out, this leads to a two-tiered provision of care: Those who can afford care as a market commodity will have access to customised care services, while all others will be left without. For the latter, the care crisis remains unresolved.

The moralisation of the domestic care market is also problematic in the sense that the moral claims brought forward in advertising campaigns do not yet translate into decent work in practice. Working conditions still undercut minimal standards in comparable sectors of the labour market by far.^7^ Intermediaries’ moralising narratives can thus be framed as a sort of ‘social washing’, capitalising on morality without significantly changing the underlining issues (Bosvieux-Onyekwelu and Boussard, 2022). Nevertheless, the moralising narratives might add pressure on regulatory bodies to improve working conditions in the care sector. A recent study shows that some labour market intermediaries, specifically in long-term care, now demand tighter regulation of their activities and enforcement by an authoritative body themselves (Mercille and Lolich, 2024). We remain cautious, as research underlines that such intermediaries typically lobby for regulations that serve the development of their for-profit firms and open up markets for their services (Ledoux et al., 2021). However, public attention to the working conditions of domestic care workers might help pave the way for regulatory interventions. In this regard, our results point to the essential role of the state and its responsibility to regulate the market, setting minimal labour standards and ensuring enforcement in practice.

## Conclusion: Increasing the collective visibility of domestic care workers

We scrutinised the ambivalent nature of moralising narratives of labour market intermediaries in the sector of domestic care work by interviewing founders and managing directors of companies who mediate domestic care workers in Switzerland, showing how they position themselves as moral entrepreneurs. They highlight their contributions to formalising domestic care work, to improving the societal recognition of care work, to emancipating women and to integrating migrants. On the one hand, we discussed the problematic aspects of these narratives in that they reify the feminisation, the migrantisation and the neoliberalisation of domestic care work. On the other hand, however, we argued that their moralising narratives also serve to draw public attention to domestic care workers and their working conditions.

The latter captures the key contribution we want to make to the existing literature: As outlined above, many scholars have argued that the widespread informality and their workspace in the private household makes domestic care workers largely invisible in labour statistics, public space and political debates. Ticona and Mateescu (2018) point out that this has changed with the advent of digital labour platforms that require workers to set up online profiles with pictures and personal information. They interpret this as a new problematic *individualised visibility* that exposes domestic care workers to the public eye without improving their work conditions. Based on the moralising narratives of the founders of such labour market intermediaries in Switzerland, we argue that this increased visibility does not necessarily remain on the level of the individual. We also find evidence of an increasing *collective visibility* of domestic care workers: entrepreneurs use labour conditions and recognition of domestic care work as advertising slogans. We are aware that discourses do not automatically enact change in practice. In our case, this means that moralising narratives do not per se translate into better working conditions. However, we should not underestimate the power of such discourses that signal the desire for change because they contribute to bringing labour conditions in domestic care work on the public agenda. The question of how moral narratives concretely shape both working and living contexts of domestic care workers in practice remains an important and pressing concern for further research.

For now, we conclude that the current moralising discourse is thus a tool that can be used to draw public attention to decent work. It strengthens already existing worker-led mobilisation for improving the conditions in the care sector. To enact significant change, it is important that it is not left to the advertising campaigns of moral entrepreneurs but is made visible as a responsibility of the state to provide care and ensure decent working conditions for those who do the work.

## Notes

1. Christiane Meyer-Habighorst conducted seven interviews in the context of the project ‘Urban Platform Economies: Transformations of Labour and Intersectional Inequalities in Care Services (TICS)’. TICS is a three-year project, running from 2022 to 2025, by Sybille Bauriedl and Me-Linh Riemann from the Europa-Universität Flensburg (Germany), Anke Strüver and Janne Martha Lentz from the University of Graz (Austria) as well as Karin Schwiter and Christiane Meyer-Habighorst from the University of Zurich (Switzerland). It is funded by the German Research Foundation, the Austrian Science Fund, and the Swiss National Science Foundation (grant number 206161). See more: https://www.uni-flensburg.de/integrative-geographie/integrative-geographie-1/forschung/tics.

2. Christina Mittmasser conducted seven interviews within the project ‘Hypervulnerability and Agency: The Impact of the Covid-19 Pandemic on Migrant Women Working in the Domestic Services Sector’. It is led by Myrian Carbajal from the University of Applied Sciences in Fribourg and Milena Chimienti from the University of Applied Sciences in Geneva. It is funded by the Swiss Science Foundations and runs from 2023 to 2026 (grant number 210024). See more: https://www.nfp80.ch/en/hcexW6SOQyYRrfm0/project/impact-of-covid-19-on-the-agency-of-migrant-women-working-in-domestic-services.

3. While we use pseudonyms for the companies of our interviewees to protect their privacy, we refer to the real name of this platform, as we drew the material from public sources. For consistency, direct quotes from the Batmaid podcast and website are numbered 15. This is because the 14 interviews we conducted and the corresponding websites are numbered from 1 to 14.

4. We have translated the quotes used in this article from interviews and website texts that were not originally in English.

5. Many of the interviewees of our study as well as Batmaid typically use the German term ‘Schwarzarbeit’ or ‘Schwarzmarkt’. Literally, this translates to ‘black labour’ or ‘black market’. To avoid racialised terminology, we translate it as ‘informal work’ and ‘informal market’ instead.

6. A childminder is a person who takes care of other people’s children, sometimes in addition to their own, in their own home and is paid to do so.

7. The mentioned platform Batmaid is an example: While the platform has offered to directly employ domestic workers since 2021 (Blick, 2020; NZZ, 2020), clients still have the option to only use the placement service and set working conditions themselves. Moreover, Batmaid had to appear before the labour court in Lausanne several times. In May 2024, for example, it was accused for the dismissal of a 2-year employee without notice. Her employment contract had only guaranteed 4 hours of work per week, but the number of hours actually worked and thereby also her salary varied greatly. With an hourly wage of 24 Swiss francs, she sometimes earned 1600 Swiss francs a month, sometimes not even half that. According to the prosecution, this is also the result of systematic violations of the collective labour agreement for the cleaning industry: Batmaid did not count the travel time between the households the worker was assigned to clean as working time, nor was there any compensation for the transport costs (Work, 2024).

## References

[bibr1-0308518X251377078] Ally S (2005) Caring about care workers: Organizing in the female shadow of globalization. Labour, Capital and Society 38(1/2): 184–207.

[bibr2-0308518X251377078] Ariztia T Kleine D Bartholo R , et al. (2016) Beyond the ‘deficit discourse’: Mapping ethical consumption discourses in Chile and Brazil. Environment and Planning A: Economy and Space 48(5): 891–909.

[bibr3-0308518X251377078] Arvidsson A (2014) Public brands and the entrepreneurial ethics. Ephemera: Theory & Politics in Organization 14(1): 119–124.

[bibr4-0308518X251377078] Aulenbacher B Lutz H Palenga-Möllenbeck E , et al. (2024) Senior home care for sale: Agency-brokered transnational live-in care in Europe. In: Aulenbacher B Lutz H Palenga-Möllenbeck E , et al. (eds) Home Care for Sale. The Transnational Brokering of Senior Care in Europe. London: Sage, pp.1–19.

[bibr5-0308518X251377078] Bachmann S (2016) Diskurse über MigrantInnen in Schweizer Integrationsprojekten. Wiesbaden: Springer.

[bibr6-0308518X251377078] Balsiger P (2021) The dynamics of ‘moralized markets’: A field perspective. Socio-Economic Review 19(1): 59–82.

[bibr7-0308518X251377078] Balsiger P Schiller-Merkens S (2019) Moral struggles in and around markets. In: Schiller-Merkens S Balsiger P (eds) The Contested Moralities of Markets. Bingley: Emerald Publishing, pp.3–26.

[bibr8-0308518X251377078] Bandinelli C (2017) Social entrepreneurship: Sociality, ethics, and politics. PhD Thesis, Goldsmiths University of London, London.

[bibr9-0308518X251377078] Barnett C Cloke P Clarke N , et al. (2011) Globalizing Responsibility: The Political Rationalities of Ethical Consumption. Chichester: Wiley-Blackwell.

[bibr10-0308518X251377078] Basellandschaftliche Zeitung (2021) Integration durch Selbstständigkeit: Migrantische Frauen gründen in Basel eigenes Reinigungsunternehmen. Available at: https://www.bzbasel.ch/basel/arbeitsmarkt-integration-durch-selbststaendigkeit-migrantische-frauen-gruenden-in-basel-eigenes-reinigungsunternehmen-ld.2218822?reduced=true (accessed 31 July 2024).

[bibr11-0308518X251377078] Batmaid (2024) About us. Available at: https://batmaid.ch/en/about-us (accessed 31 July 2024).

[bibr12-0308518X251377078] Bereni L (2023) Le management de la vertu: La diversité en entreprise à New York et à Paris. Paris: Presses de Sciences Po.

[bibr13-0308518X251377078] Berg J Furrer M Harmon E , et al. (2018) Digital Labour Platforms and the Future and the Future of Work: Towards Decent Work in the Online World. Geneva: ILO International Labour Organization.

[bibr14-0308518X251377078] Blick (2020) Hingis-Firma kriegt auf den Putz. Batmaid muss Putzpersonal nun doch anstellen. 20 July. Available at: https://www.blick.ch/wirtschaft/hingis-firma-kriegt-auf-den-putz-batmaid-muss-putzpersonal-nun-doch-anstellen-id16002177.html (accessed 31 July 2024).

[bibr15-0308518X251377078] Blick (2021) Reinigungsbranche boomt – doch längst nicht alle Anbieter sind sauber. Jede vierte Putzkraft arbeitet schwarz. Available at: https://www.blick.ch/wirtschaft/reinigungsbranche-boomt-doch-laengst-nicht-alle-anbieter-sind-sauber-jede-vierte-putzkraft-arbeitet-schwarz-id16348319.html (accessed 31 July 2024).

[bibr16-0308518X251377078] Bosvieux-Onyekwelu C Boussard V (2022) Moraliser le capitalisme ou capitaliser sur la morale? Actes de la recherche en sciences sociales 241(1): 4–15.

[bibr17-0308518X251377078] Brenner N Peck J Theodore N (2010) Variegated neoliberalization: Geographies, modalities, pathways. Global Networks 10(2): 182–222.

[bibr18-0308518X251377078] Bundesamt für Sozialversicherungen (2025) Finanzhilfen für die Schaffung von Betreuungsplätzen. Available at: https://www.bsv.admin.ch/bsv/de/home/finanzhilfen/kinderbetreuung/finanzhilfen-schaffung-betreuungsplaetze.html (accessed 28 May 2025).

[bibr19-0308518X251377078] Buttle M (2008) Diverse economies and the negotiations and practices of ethical finance: the case of Charity Bank. Environment and Planning A: Economy and Space 40(9): 2097–2113.

[bibr20-0308518X251377078] Cox R (2013) Gendered spaces of commoditised care. Social & Cultural Geography 14(5): 491–499.

[bibr21-0308518X251377078] Credit Suisse (2022) Global Wealth Report. Zurich: Credit Suisse Research Department.

[bibr22-0308518X251377078] Daniels AK (1987) Invisible work. Social Problems 34(5): 403–415.

[bibr23-0308518X251377078] Dey P (2010) The symbolic violence of ‘social entrepreneurship’: Language, power and the question of the social (subject). In: Third research colloquium on social entrepreneurship, Oxford, UK, 22–25 June.

[bibr24-0308518X251377078] Dey P (2014) Governing the social through ‘social entrepreneurship’: A Foucauldian view of the ‘art of governing’ in advanced liberalism. In: Douglas H Grant S (eds) Social Innovation and Social Entrepreneurship: Context and Theories. Melbourne: Tilde University Press, pp.55–72.

[bibr25-0308518X251377078] Döringer S (2020) ‘The problem-centred expert interview’. Combining qualitative interviewing approaches for investigating implicit expert knowledge. International Journal of Social Research Methodology 24(3): 265–278.

[bibr26-0308518X251377078] Dowling E (2021) The Care Crisis: What Caused It and How Can We End It? London: Verso.

[bibr27-0308518X251377078] Ecker Y Rowek M Strüver A (2021) Care on demand: Geschlechternormierte Arbeits- und Raumstrukturen in der plattformbasierten Sorgearbeit. In: Altenried M Dück J Wallis M (eds) Plattformkapitalismus und die Krise der sozialen Reproduktion. Münster: Westfälisches Dampfboot, pp.112–129.

[bibr28-0308518X251377078] Farris SR Horton A Lloyd E (2024) Corporatisation and financialisation of social reproduction: Care homes and childcare in the United Kingdom. Environment and Planning F: Philosophy, Theory, Models, Methods and Practice 4(3): 340–356.

[bibr29-0308518X251377078] Fetterolf E (2022) It’s crowded at the bottom: Trust, visibility, and search algorithms on Care.com. Journal of Digital Social Research 4(1): 49–72.

[bibr30-0308518X251377078] Folbre N (2001) The Invisible Heart: Economics and Family Values. New York, NY: New Press.

[bibr31-0308518X251377078] Foucault M (1988) Technologies of the self. In: Martin L Gutman H Hutton P (eds) Technologies of the Self: A Seminar with Michel Foucault. Amherst: University of Massachusetts Press, pp.16–49.

[bibr32-0308518X251377078] Foucault M (1991) Governmentality. In: Burchell G Gordon C Miller P (eds) The Foucault Effect: Studies in Governmentality. Hemel Hempstead: Harvester Wheatsheaf, pp.87–104.

[bibr33-0308518X251377078] Fraser N (2017) Crisis of care? On the social-reproductive contradictions of contemporary capitalism. In: Bhattacharya T (ed.) Social Reproduction Theory: Remapping Class, Recentering Oppression. London: Pluto Press, pp.21–36.

[bibr34-0308518X251377078] Geiger S Harrison D Kjellberg H , et al. (2014) Concerned Markets: Economic Ordering for Multiple Values. Northampton: Edward Elgar.

[bibr35-0308518X251377078] Glaser BG Strauss AL (1967) The Discovery of Grounded Theory: Strategies for Qualitative Research. New Brunswick: Aldine Transaction.

[bibr36-0308518X251377078] Green M Lawson V (2011) Recentring care: Interrogating the commodification of care. Social & Cultural Geography 12(6): 639–654.

[bibr37-0308518X251377078] Griffiths M Santos Rodriguez V (2022) Reclaiming Agency through the Politics of the In_Visible Body: Illegalized migration and self-representation of women domestic workers in Switzerland. The Open Journal for the Study of Culture 13: 1–34.

[bibr38-0308518X251377078] Gruszka K Böhm M (2022) Out of sight, out of mind? (In)visibility of/in platform-mediated work. New Media & Society 24(8): 1852–1871.

[bibr39-0308518X251377078] Gutiérrez Rodriguez E (2011) Migration, Domestic Work and Affect: A Decolonial Approach on Value and the Feminization of Labor. New York, NY; London: Routledge.

[bibr40-0308518X251377078] Hall S (2001) Foucault: Power, knowledge and discourse. In: Wetherell M Taylor S Yates SJ (eds) Discourse Theory and Practice: A Reader. London: Sage, pp.72–81.

[bibr41-0308518X251377078] Harvey D (2005) A Brief History of Neoliberalism. Oxford: Oxford University Press.

[bibr42-0308518X251377078] Hatton E (2017) Mechanisms of invisibility: Rethinking the concept of invisible work. Work, Employment and Society 31(2): 336–351.

[bibr43-0308518X251377078] Hochschild AR (1997) The Time Behind: When Work Becomes Home and Home Becomes Work. New York, NY: Metropolitan Books.

[bibr44-0308518X251377078] Hunt A Machingura F (2016) A Good Gig? The Rise of On-Demand Domestic Work. London: Overseas Development Institute.

[bibr45-0308518X251377078] Huws U (2019) The hassle of housework: Digitalisation and the commodification of domestic labour. Feminist Review 123(1): 8–23.

[bibr46-0308518X251377078] International Labour Organization (2013) Domestic Workers Across the World: Global and Regional Statistics and the Extent of Legal Protection. Geneva: International Labour Office.

[bibr47-0308518X251377078] James A (2024) Platform work-lives in the gig economy: Recentering work–family research. Gender, Work & Organization 31(2): 513–534.

[bibr48-0308518X251377078] Kelpin R (2020) Morality on tap: The production and consumption of morality by ‘Vegandale’. Canadian Journal of Urban Research 29(2): 55–69.

[bibr49-0308518X251377078] Kish Z Fairbairn M (2018) Investing for profit, investing for impact: Moral performances in agricultural investment projects. Environment and Planning A: Economy and Space 50(3): 569–588.

[bibr50-0308518X251377078] Kofman E Raghuram P (2015) Gendered Migrations and Global Social Reproduction. London: Palgrave Macmillan.

[bibr51-0308518X251377078] Kortelainen J (2008) Performing the green market – Creating space: Emergence of the green consumer in the Russian woodlands. Environment and Planning A: Economy and Space 40(6): 1294–1311.

[bibr52-0308518X251377078] Laugier S (2021) Paradoxes in the invisibility of care work. Philosophical Topics 49(1): 61–80.

[bibr53-0308518X251377078] Ledoux C Encinas de Muñagorri R Guiraudon V (2021) Becoming an organised actor in a welfare market: Employers in the French in-home domestic/care services sector. In: Ledoux C Shire K van Hooren F (eds) The Dynamics of Welfare Markets. Work and Welfare in Europe. Cham: Palgrave Macmillan, pp.319–344.

[bibr54-0308518X251377078] Macdonald F Bentham E Malone J (2018) Wage theft, underpayment and unpaid work in marketised social care. The Economic and Labour Relations Review 29(1): 80–96.

[bibr55-0308518X251377078] McDonald P Williams P Mayes R , et al. (2024) Income generation on care work digital labour platforms. British Journal of Industrial Relations 62(2): 358–380.

[bibr56-0308518X251377078] McEwan C Hughes A Bek D (2017) Fairtrade, place and moral economy: Between abstract ethical discourse and the moral experience of Northern Cape farmers. Environment and Planning A: Economy and Space 49(3): 572–591.

[bibr57-0308518X251377078] Mercille J (2024) European long-term care marketisation: A political economy framework. Social Policy & Administration 58(7): 1059–1074.

[bibr58-0308518X251377078] Mercille J Lolich L (2024) What do private providers of home care want? An analytical framework. Critical Public Health 34(1): 1–14.40182057

[bibr59-0308518X251377078] Mexi MM Moreno Russi P Guzman EF (2021) ‘Fortress’ Switzerland? Challenges to integrating migrants, refugees and asylum-seekers. In: Federico V Baglioni S (eds) Migrants, Refugees and Asylum Seekers’ Integration in European Labour Markets. Cham: Springer, pp.213–233.

[bibr60-0308518X251377078] Millet A (2023) Woke Washing: Capitalisme, Consumérisme, Opportunisme. Paris: Les Pérégrines.

[bibr61-0308518X251377078] Mittmasser C Stingl I (2021) Migrant counterspaces: Challenging labour market exclusion through collective action. Revue européenne des migrations internationales 37(1/2): 229–249.

[bibr62-0308518X251377078] Nadai E Canonica A (2019) The moralization of labor: Establishing the Social responsibility of employers for disabled workers. In: Schiller-Merkens S Balsiger P (eds) The Contested Moralities of Markets. Bingley: Emerald Publishing, pp.87–106.

[bibr63-0308518X251377078] Näre L (2011) The moral economy of domestic and care labour: Migrant workers in Naples, Italy. Sociology 45(3): 396–412.

[bibr64-0308518X251377078] Neue Zürcher Zeitung (2020) Batmaid stellt ihre Reinigungshilfen jetzt an. Available at: https://www.nzz.ch/wirtschaft/batmaid-will-reinigungshilfen-fest-anstellen-ld.1791448 (accessed 31 July 2024).

[bibr65-0308518X251377078] Neue Zürcher Zeitung (2021) Nie mehr für 16 Franken pro Stunde putzen: Eine Gruppe von Reinigungskräften in Zürich will faire Arbeitsbedingungen und gründet ihre eigene Vermittlungsplattform. Available at: https://www.nzz.ch/wirtschaft/fuer-fairere-arbeitsbedingungen-zuercher-reinigungskraefte-gruenden-ihre-eigene-vermittlungsplattform-ld.1661509?reduced=true (accessed 31 July 2024).

[bibr66-0308518X251377078] Organisation for Economic Co-operation and Development (2023) Long-Term Care Spending and Unit Costs. Paris: OECD Publishing.

[bibr67-0308518X251377078] Organisation for Economic Co-operation and Development (2025) Net childcare costs. Available at: https://www.oecd.org/en/data/indicators/net-childcare-costs.html (accessed 28 May 2025).

[bibr68-0308518X251377078] Orth B (2024) Stratified pathways into platform work: Migration trajectories and skills in Berlin’s gig economy. Environment and Planning A: Economy and Space 56(2): 476–490.

[bibr69-0308518X251377078] Pandey K Parreñas RS Sabio GS (2021) Essential and expendable: Migrant domestic workers and the COVID-19 pandemic. American Behavioral Scientist 65(10): 1287–1301. 38603104 10.1177/00027642211000396 PMC7969853

[bibr70-0308518X251377078] Parreñas R (2001) Servants of Globalisation: Women, Migration, and Domestic Work. Stanford, CA: Stanford University Press.

[bibr71-0308518X251377078] Peck J Tickell A (2002) Neoliberalizing space. Antipode 34(3): 380–404.

[bibr72-0308518X251377078] Rathi A Tandon A (2021) Platforms, Power, & Politics. Perspectives from Domestic & Care Work in India. Bengaluru; New Delhi: Centre for Internet and Society.

[bibr73-0308518X251377078] Rodríguez-Modroño P Agenjo-Calderón A López-Igual P (2022) Platform work in the domestic and home care sector: New mechanisms of invisibility and exploitation of women migrant workers. Gender & Development 30(3): 619–635.

[bibr74-0308518X251377078] Rosenman E Loomis J Cohen D , et al. (2024) Bringing life’s work to market: Frontiers, framings, and frictions in marketised social reproduction. Environment and Planning A: Economy and Space 56(1): 190–198.

[bibr75-0308518X251377078] Schilliger S (2024) Breaking out of the ‘prisoner of love’ dilemma: Infrastructures of solidarity for live-in care workers in Switzerland. In: Aulenbacher B Lutz H Palenga-Möllenbeck E , et al. (eds) Home Care for Sale. The Transnational Brokering of Senior Care in Europe. London: Sage, pp.278–292.

[bibr76-0308518X251377078] Schilliger S Schwiter K Steiner J (2023) Care crises and care fixes under Covid-19: The example of transnational live-in care work. Social & Cultural Geography 24(3/4): 391–408.

[bibr77-0308518X251377078] Schinkel W (2018) Against ‘immigrant integration’: For an end to neocolonial knowledge production. Comparative Migration Studies 6(1): 31. 30363772 10.1186/s40878-018-0095-1 PMC6182342

[bibr78-0308518X251377078] Schwiter K Berndt C Truong J (2018) Neoliberal austerity and the marketization of elderly care. Social & Cultural Geography 19(3): 379–399.

[bibr79-0308518X251377078] Schwiter K Steiner J (2020) Geographies of care work: The commodification of care, digital care futures and alternative caring visions. Geography Compass 14(12): e12546.

[bibr80-0308518X251377078] Shamir R (2008) The age of responsibilization: On market-embedded morality. Economy and Society 37(1): 1–19.

[bibr81-0308518X251377078] SRF (2022) ‘Die Idee’: Basler Migrantinnen gründen eigene Firma. Available at: https://www.srf.ch/play/tv/10-vor-10/video/die-idee-basler-migrantinnen-gruenden-eigene-firma?urn=urn:srf:video:ee51031f-690e-4aa7-9e10-262996dedcaf (accessed 31 July 2024).

[bibr82-0308518X251377078] Strauss K Xu F (2018) At the intersection of urban and care policy: The invisibility of eldercare workers in the global city. Critical Sociology 44(7/8): 1163–1187.

[bibr83-0308518X251377078] Suckert L (2018) Unravelling ambivalence: A field-theoretical approach to moralised markets. Current Sociology 66(5): 682–703.

[bibr84-0308518X251377078] Swisspreneur (2023) EP #322 – Andreas Schollin-Borg: How to run a people business. Available at: https://www.swisspreneur.org/podcast/andreas-schollin-borg-ep322 (accessed 31 July 2024).

[bibr85-0308518X251377078] Tages-Anzeiger (2022) Putzjobs für Eingewanderte – Sprungbrett oder Sackgasse. Available at: https://www.tagesanzeiger.ch/putzjobs-fuer-eingewanderte-sprungbrett-oder-sackgasse-234222838551 (accessed 31 July 2024).

[bibr86-0308518X251377078] Tages-Anzeiger (2024) Die Putzfrauen, die sich selbst zur Chefin gemacht haben. Available at: https://www.tagesanzeiger.ch/reinigung-in-zuerich-und-basel-putzfrauen-machen-sich-selbst-zur-chefin-122736915751 (accessed 31 July 2024).

[bibr87-0308518X251377078] Ticona J (2022) Left to Our Own Devices: Coping with Insecure Work in a Digital Age. Oxford: Oxford University Press.

[bibr88-0308518X251377078] Ticona J Mateescu A (2018) Trusted strangers: Carework platforms’ cultural entrepreneurship in the on-demand economy. New Media & Society 20(11): 4384–4404.

[bibr89-0308518X251377078] United Nations International Children’s Emergency Fund (2021) Where Do Rich Countries Stand on Childcare? Florence: UNICEF Office of Research – Innocenti.

[bibr90-0308518X251377078] Van Doorn N (2022) Platform capitalism’s social contract. Internet Policy Review 1(1): 1–18.

[bibr91-0308518X251377078] Van Doorn N (2023) Liminal precarity and compromised agency: Migrant experiences of gig work in Amsterdam, Berlin, and New York City. In: Ness I (ed.) The Routledge Handbook of the Gig Economy. London; New York, NY: Routledge, pp.158–179.

[bibr92-0308518X251377078] Waitt G (2016) Doing Foucauldian discourse analysis: Revealing social realities. In: Hay I (ed.) Qualitative Research Methods in Human Geography. Oxford; New York, NY: Oxford University Press, pp.289–312.

[bibr93-0308518X251377078] Wochenzeitung (2022) Was hat Sie am Genossenschaftsmodell überzeugt? Available at: https://www.woz.ch/2205/durch-den-monat-mit-adele-villiger-teil-1/was-hat-sie-am-genossenschaftsmodell-ueberzeugt (accessed 31 July 2024).

[bibr94-0308518X251377078] Wochenzeitung (2024) Arbeit auf Abruf. Unterm Glanz der Oberfläche. Available at: https://www.woz.ch/2407/arbeit-auf-abruf/unterm-glanz-der-oberflaeche/!HRB4QDAVTV21 (accessed 31 July 2024).

[bibr95-0308518X251377078] Wolford W (2005) Agrarian moral economies and neoliberalism in Brazil: Competing worldviews and the state in the struggle for land. Environment and Planning A: Economy and Space 37(2): 241–261.

[bibr96-0308518X251377078] Work (2024) Dumping-Prozess: Batmaid-Boss rastet aus – und muss blechen. Reinigering wäscht Putz-CEO die Kappe. Available at: https://www.workzeitung.ch/2024/05/reinigerin-waescht-putz-ceo-die-kappe/ (accessed 31 July 2024).

[bibr97-0308518X251377078] Zelizer VA (1979) Morals and Markets. The Development of Life Insurance in the United States. New York, NY: Columbia University Press.

